# Clovibactin and Staphylococcus aureus: a new weapon against resistant strains

**DOI:** 10.3205/dgkh000501

**Published:** 2024-10-23

**Authors:** Shuaibu Suleiman Adeiza

**Affiliations:** 1 Department of Pharmaceutical Microbiology and Biotechnology, Faculty of Pharmaceutical Sciences, Ahmadu Bello University, Zaria, Kaduna, Nigeria; 2 Department of Clinical Pharmacy and Pharmacy Practice, Usmanu Danfodiyo University, Sokoto, Nigeria

**Keywords:** clovibactin, Staphylococcus aureus, antimicrobial resistance, Eleftheria terrae

## Abstract

Clovibactin is a new depsipeptide and highly efficacious against *Staphylococcus* (*S.*) *aureus*, including methicillin-resistant and vancomycin-resistant *S.* *aureus*, with no apparent resistance. Clovibactin outclasses current antibiotics such as vancomycin. Here, we discuss its efficacy, emphasize the need for new antibiotics owing to growing global antibiotic resistance, highlight its mode of action and possible benefits over current treatments. We also highlight the challenges involved in large-scale manufacturing and the status of continuing research to advance effective and less toxic derivatives.

## Abstract (de)

Clovibactin ist ein neues Depsipeptid und hoch wirksam gegen *Staphylococcus* (*S.*) *aureus*, einschließlich Methicillin-resistentem und Vancomycin-resistentem *S.* *aureus* ohne erkennbare Resistenz. Clovibactin übertrifft die derzeitigen Antibiotika wie Vancomycin. Wir erörtern hier die Wirksamkeit, den dringenden Bedarf an neuen Antibiotika aufgrund der weltweit zunehmenden Antibiotikaresistenz, den Wirkungsmechanismus und mögliche Vorteile gegenüber den derzeitigen Behandlungen. Außerdem werden die Probleme bei der Herstellung in großem Maßstab und der Stand der Forschung zur Entwicklung wirksamer und weniger toxischer Derivate beleuchtet.

## Introduction

Antibiotics may be classified based on the part of the cell they affect, also based on whether they cause cell inhibition (bacteriostatic) or death (bactericidal) [1]. The majority of bactericidal drugs inhibit synthesis of either the cell wall, DNA, RNA, or protein [1]. Cell death caused by antibiotics is a process that starts with a drug molecule interacting with its target bacteria, followed by biochemical, molecular, and ultrastructural changes in the target [2]. The ability of antibiotics to kill bacteria usually involves blocking DNA gyrase, which promotes the breakage of double-stranded DNA, stopping DNA synthesis, and damaging the cell envelope. Additionally, blocking cell wall formation results in the destruction of cell wall stability [3], [4]. Certain antibiotics interfere with translation, causing protein synthesis errors and increasing cellular energy demand for stress management. This diverts resources from other processes, reducing efficiency and raising energy consumption [3], [4]. In the case of drug resistance, the actions of antibiotics mentioned above, including killing or inhibiting bacteria by targeting the cellular processes, will fail [5].

After the golden age of antibiotic discovery (1940s–1960s), the field faced great challenges in the decades that followed, and no new antibiotics were developed [6]. Antibiotic resistance killed about 1,270,000 people world-wide in 2019 and has since contributed to four times more deaths as resistance continues to rise globally [7]. In WHO African regions, bacteria-associated Antimicrobial resistance (AMR) deaths were estimated to be 1.05 million: of these 250,000 were linked directly to AMR in 2019 [8]. Over 100,000 of these deaths were linked to *S. aureus* (MRSA was the chief culprit) [8]. By 2050, AMR could result in over 10 million deaths annually, outstripping cancer as the leading cause of death [9].

*Staphylococcus (S.) aureus*, chiefly those strains that are resistant to vancomycin (VRSA) and methicillin (MRSA), is ranked among the top six most menacing drug-resistant bacteria for which newer antibiotics are needed [10]. *S*. *aureus* is harmful and causes infections in both soft tissue and skin [11]. Left unattended, these infections can result in serious conditions such as bacteraemia or septic shock, with significant death rates [12]. In people aged 15 and older, *S. aureus* is the leading cause of bacteria-associated deaths globally [12]. In 2019, around 569,000 mortalities were attributed to antibiotic resistant bacteria in the Americas (WHO-regions), with a confidence interval between 406,000 and 771,000 [13]. Furthermore, 141,000 mortalities were directly triggered by these bacteria, with the spectrum projected to be in the range of 99,900 to 196,000 [13].

**Figure 1:**
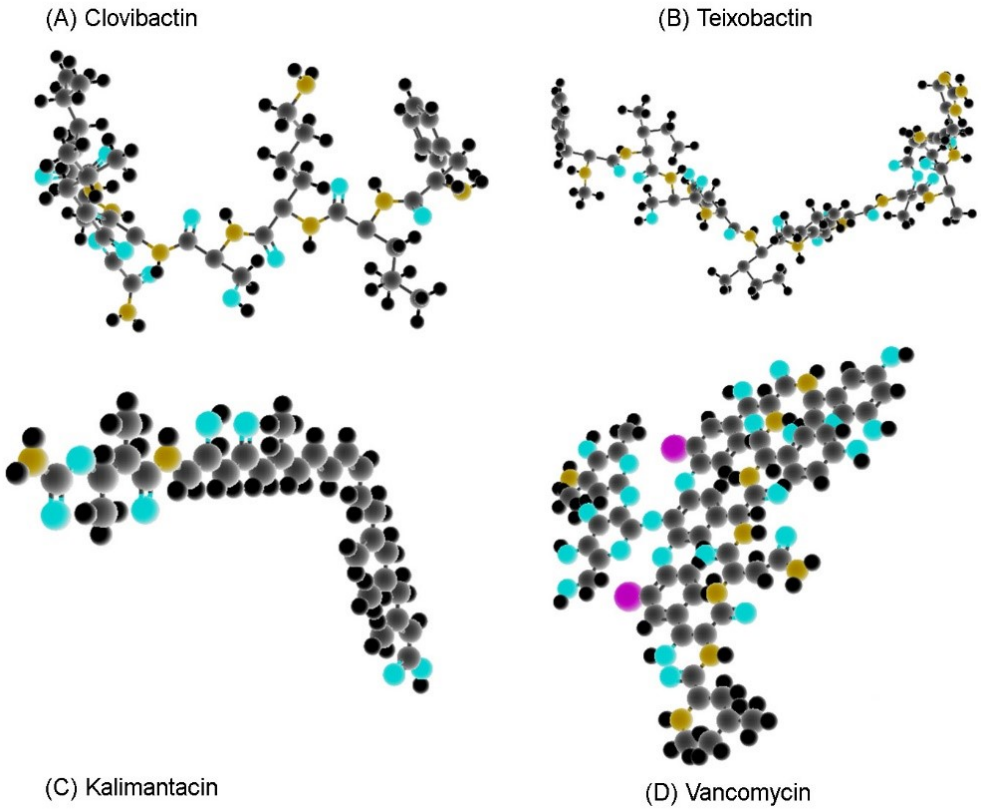
Three-dimensional structure of clovibactin, teixobactin, kalimantacin and vancomycin

Today, innovative technologies, e.g., machine learning, quantum computing and next-generation sequencing, are speeding up the identification of bacterial drug resistance and helping us to better understand the genes responsible for them [14]. Scientists are also working on novel treatments, for instance, antimicrobial peptides, phage therapy, vaccines, treatments that target the host’s own cells, and photodynamic therapy [10]. But treatment of diseases/infections caused by bacteria still largely consists of antibiotics. Excessive use of these drugs has led to a strong upsurge in drug-resistant strains such as MRSA [15]. MRSA infections are worse than methicillin susceptible strains , more expensive to treat, and require longer hospital stays [16]. MRSA causes many HAIs (hospital-acquired infections) and the last resort-antibiotic, vancomycin, is failing with increasing frequency [17]. The importance of finding new antibiotics cannot be overstated [18]. In a 2023 breakthrough by Shukla et al., clovibactin was discovered (Figure 1 (Fig. 1)). Using the isolation chip or iChip device, it was isolated from an uncultured bacterium [19]. Markus Weingarth et al. at Utrecht University in the Netherlands have studied clovibactin and found that it outperforms vancomycin as a potent MRSA killer [19].

Clovibactin is an antibiotic that kills Gram-positive pathogenic, drug-resistant bacteria [20]. It was harvested from an uncultured bacterium found in the soil [19]. No resistance to it has yet been found [21]. This compound targets the pyrophosphate of the precursors of peptidoglycan (PG) (lipid II, lipid IIIWTA and C55PP), thereby blocking the cell wall synthesis of organisms of interest [22]. This mode of action was confirmed using atomic force microscopy, nuclear magnetic resonance (solid state) and biochemical tests [23]. It accounts for lack of resistance by using a rare hydrophobic interphase that wraps tightly around pyrophosphate and avoids variable precursor structural elements [23]. For some bacterial membranes that have lipid groups with pyrophosphate, this antibiotic achieves specificity and efficiency for effective bacteriocide by forming supramolecular fibril structures [24]. This technique helps in creation of better drugs with less potential for resistance developing in its target [24]. 

The purpose of this review is to describe clovibactin as a novel antibiotic that is effective against *S. aureus* and its drug-resistant strains, including MRSA, and emphasize the pressing requirement for innovative treatment options with new antibiotics like clovibactin and its analogues to fight resistance to antibiotics.

## Discovery of clovibactin from Eleftheria terrae

**Figure 2:**
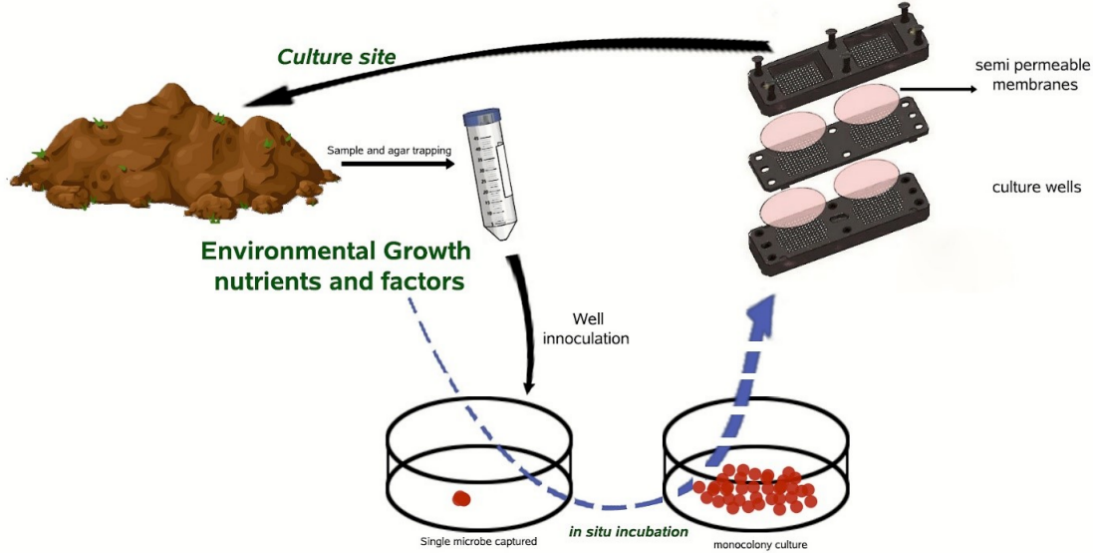
Cultivation of bacteria using the iChip device

The isolation chip or iChip device (Figure 2 (Fig. 2)) helps researchers cultivate bacteria that were formerly difficult to grow [25]. It functions by taking bacterial samples, trapping them in microwells on the chip, and then returning the chip to its natural habitat so that the bacteria can flourish [25]. This system has brought about a massive rise (30,000%) in microbial growth when compared to traditional methods [26]. Using the iChip, bacteria can be cultivated from vast sources, e.g., soil, seawater, wastewater and saliva [27]. This technique has led scientists to discover over 25 new antibiotics and 10,000 novel microbial species [26]. Of these new antibiotics, clovibactin was found in a recently discovered organism termed *Eleftheria terrae* [22]. Clovibactin is promising, as it can kill extremely drug-resistant bacteria and it has proved to protect mice from MRSA [21]. The iChip is a hard plastic chip with nearly 200 wells [26]. The unit is dipped into an agar laden with the bacterial sample in order to ensure that every well contains a cell [26]. A diffusion membrane is positioned on the lateral sides of the chip and held with plastic tape to fix the bacteria in place [26], [28]. The iChip, with its sample is placed back into the bacterium’s natural habitat (soil, bodies of water, etc.) where nutrients may pass through the diffusion membrane [26]. This diffusion of growth factors across semi-permeable membranes supports uncultured bacteria growth (nearly 99% of all uncultured bacteria, in their natural habitat) [28].

*Eleftheria *(*E.*) *terrae* belongs to the Eleftheria family, which includes the producer of another recently discovered antibiotic, teixobactin, and was isolated from soil in North Carolina (USA) [29]. Colonies of this bacterium detected after 2.76 months of incubation were sub-cultured on nutrient agar plates spread with *S. aureus* and screened for antimicrobial activity [19]. A clear zone of inhibition was observed around the *E. terrae*. Fractions of the extracts as identified by biological assays led to the isolation of a new depsipeptide molecule called NOVO-29 (clovibactin) [19], [30]. 

*E. terrae* is capable of making more than one antibiotic (clovibactin, teixobactin and kalimantacin) [19]. By means of high-pressure liquid chromatography (HPLC), part of the mixture capable of killing *S. aureus* and *B. subtilis* was identified and the genes of *E. terrae* were altered to focus on the production of clovibactin [19]. The structure of clovibactin, as elucidated by NMR, was found to be similar to teixobactin in its molecular scaffold but with some important differences. It looks like a peptide, but has a mix of ester bonds and amino acids (hybrid) [19], [30].

## Staphylococcus aureus cell wall and antibiotics

The discovery of clovibactin has been greeted as a step forward in the search for new therapeutic alternatives. Currently, knowledge of how this compound works is advancing through preclinical testing [20].

**Figure 3:**
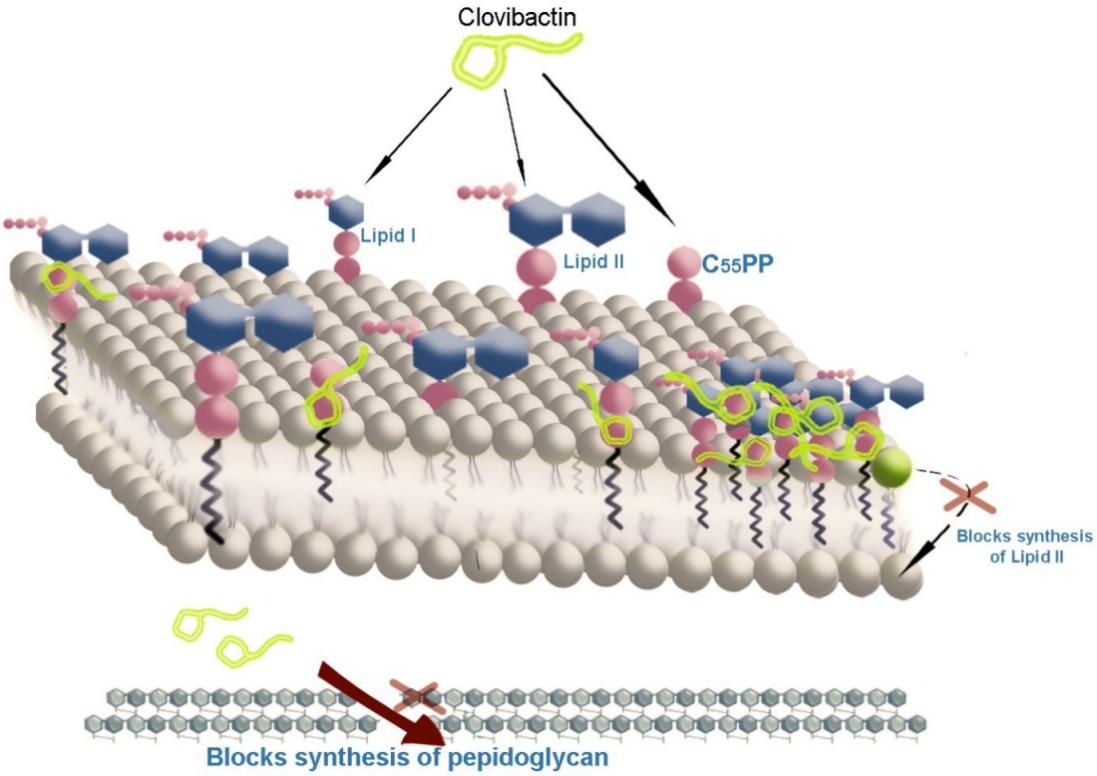
Mechanism of action. Diagrams show how clovibactin interacts with the peptidoglycan layer of *S. aureus* and inhibits cell wall synthesis.

Peptidoglycan (PG) synthesis begins with the development of a molecule termed UDP-MurNAc, which is then connected with a pentapeptide section [30]. This compound is joined with undecaprenyl pyrophosphate to make lipid I. Another molecule, UDP-GlcNAc, is joined to lipid I to produce lipid II. This entire progression happens within the cell membrane [31]. lipid II is then transported perpendicularly through the membrane, where penicillin-binding proteins aid in building and reinforcing the cell wall by linking the PG subunits [31]. The undecaprenyl pyrophosphate is returned to the cell for an additional cycle of use [31].

lipid II is susceptible to antibiotic attack when it is outside the bacterial membrane. Its design offers numerous targets for antibiotics, such as the pentapeptide domain (which vancomycin targets) or the pyrophosphate moiety targeted by other antibiotics, e.g., lantibiotics and ramoplanin [32]. The quantity of lipid II in the cell membrane is small at any point in time, as it is rapidly consumed in cell-wall formation [19]. Obstructing lipid II effectively (Figure 3 (Fig. 3)) stops the bacteria from constructing and re-inforcing their cell wall, resulting in their death [19].

The *S. aureus* cell is surrounded by strata of murein or PG, a tightly linked network composed of interconnected peptides and sugar molecules (β-(1–4)-N-acetyl hexosamine) [33]. The mechanical strength of this layer aids the *S. aureus* bacterium to survive under adverse conditions, such as fluctuations in osmotic pressure [33]. The more PG crosslinking there is in the bacterial wall, the more durable and stable the bacteria will be. This increased structural integrity and resilience makes the bacteria more resistant to external stressors and mechanical disruption [33], [34]. The integrity of the PG layer is maintained by the enzymes trans glycosidase (which adds sugar peptides to elongate strands) and transpeptidase (links elongated strands together) [33]. Because PG is crucial for maintaining the physical integrity of bacterial cell walls, it is an important target for many antibiotics, including those of the beta-lactam group and vancomycin [33].

Effective therapy with drugs that disturb the homoeostasis of cell-wall synthesis – for instance, glycopeptides and β-lactams – modifies cell structure and dimensions, causing stress inside the cell which ruptures it [23] (Figure 1b (Fig. 1)). β-lactams, such as penicillin, carbapenems and cephalosporins, prevent bacteria from assembling their cell walls [21]. They do this by attaching to a part of the enzyme that facilitates cell-wall assembly (they block PG cross-linking), disabling the enzyme (penicillin binding proteins, PBPs) and causing the bacteria to cease growing [22].

The mechanism of action of glycopeptide antibiotics like vancomycin (which is derived from actinobacteria) differs substantially from that of β-lactams. Glycopeptide antibiotics se interrupt cell-wall maintenance by seizing onto a section of the PG edifice, to be exact, the D-alanyl-D-alanine portion [22]. This seizing action stops the enzymes transpeptidase and transglycosylase, which are vital for cell-wall assembly [35]. As an effect, glycopeptides retard cell-wall construction and compromise the integrity of the cell wall [36]. Unlike β-lactams (effective on Gram-positive and Gram-negative bacteria), glycopeptides are solely effective against Gram-positive bacteria, since they are not able permeate into Gram-negative bacteria [19]. Also, other antibiotic types, such as Fosfomycin and Bacitracin, also disrupt different cell-wall maintenance processes [19].

*S. aureus* possesses a lysis-toxin sensor regulator (LytSR) that can influence cell lysis by regulating the action of enzymes that decompose cell walls (autolysins) [37]. LytSR activates genes such as the locus of reduced genome AB (LrgAB), which decelerate autolysins and increase the resistance of antibiotics [38]. LrgA regulates how enzymes access the cell wall [38]. Another system, termed “cell death-inducing cidAB” does the opposite, activating autolysins and making *S. aureus* easier to kill [19].

## Antibiotics’ mechanisms and clovibactin efficacy

Some studies have examined how antibiotics that halt cell wall construction can kill bacteria [39]. At first, they assumed that these antibiotics triggered cell death by increasing pressure inside the cell to the extent that, when it grew faster than its walls, it ruptured [40]. This was centred on the premise that protein synthesis was required for antibiotic-associated cell rupture. However, the machinery of cell death through lysis comprises active cellular processes such as poor amidase activity in, for instance, *Streptococcus pneumoniae*, leading to antibiotic tolerance. This highlights the role of autolysins in breaking down PG and contributing to lytic cell death in bacteria like *Escherichia coli* (*E. coli*), when coupled with cessation of PG synthesis by β-lactam antibiotics [41].

In susceptibility testing, the activity of clovibactin was good against *S. aureus* and its drug-resistant strains (DRSA, VISA, and MRSA) [21], [22]. It was effective against vancomycin-resistant *Enterococcus faecium* and *Enterococcus faecalis* [22], but comparatively less effective against *E. coli*, due to the poor penetration efficacy of the compound [22], [42].

## Structure, targets, and efficacy

Clovibactin is bactericidal against *S. aureus*, with an MBC (minimum bactericidal concentration) twice that of the MIC (minimum inhibitory concentration). When compared to vancomycin, its *S. aureus *killing effect is greater. Similar to teixobactin [36], it causes strong breakage of cell structure. Teixobactin molecules stick together and form pairs (hydrogen-bonded dimers). These pairs then group on the cell membrane in a β-sheet formation, causing important cell wall precursors to cluster, leading to the bacteria breaking apart. Likewise, clovibactin acts in a way that permits its side chains (Phenylalanine, Leucine and its isomer, D-Leucine) to insert into the cell membrane of *S. aureus*. Clovibactin molecules also stick together through hydrophobic interactions and hydrogen bonding, causing lipid II and related cell-wall precursors to cluster and the bacteria to break apart [22]. Furthermore, unlike teixobactin, clovibactin could still lyse the cells even in the absence of *AtlA* protein [42].

To discover the target of clovibactin, first, the resistance frequency of *S. aureus* was determined to be lower than 10^8^ (less than 10^10^ was desirable) after an antimicrobial susceptibility assay of clovibactin-impregnated (less than four times the MIC) medium with *S. aureus* [43]. There was no visible resistance, even at such a low concentration [19]. This means the likelihood of drug resistance is less than one in ten billion. Knowledge of drug targets is very crucial for drug development, and understanding how often *S. aureus* may become resistant to a given drug ensures its clinical life-span [43]. The biosynthetic pathway inhibited by clovibactin was traced using radiolabelled precursors injected into *S. aureus*. Of the major pathways of bacterial synthesis (PGs, Protein, RNA and DNA), clovibactin only prevented N-acetylglucosamine (GlcNAc) in the PG from being used in cell-wall synthesis [19].

To elucidate the specific effect of clovibactin, special tests were required to visualize temporal bacterial reactions. The *LiaRS* system responds to antibiotics that affect lipid II biosynthesis in the cell wall was utilized [44]. LiaRS is a two-component regulatory system in *S. aureus*, consisting of a sensor kinase (LiaS) and a response regulator (LiaR), that is triggered when the bacterial respond to environmental stimuli. LiaRS is specifically activated by the presence of antibiotics targeting lipid II biosynthesis. The PliaI-lux test is an assay used to detect the activation of LiaRS via a bioluminescent reporter, allowing for the detection and quantification of LiaRS activity through bioluminescence measurements. Clovibactin triggered a positive reaction in the PliaI-lux test, in which light emission signifies the activation of the *LiaI* promoter, indicative of its direct effect on the lipid II biosynthesis [22], [45].

In the *S. aureus* cytoplasm, the nucleotide sugars UDP-GlcNAc and UDP-N-acetylmuramic acid are biosynthesized [33]. These sugars are then bound to a lipid carrier known as undecaprenyl phosphate (C55P) to form lipid II [33]. This lipid is added to a branching network of PG in cell-wall synthesis. Clovibactin might work by preventing the occurrence of this later stage, resulting in the intracellular build-up UDP-GlcNAc and UDP-N-acetylmuramic acid, which will eventually lead to the death of the bacterial cell [33].

Clovibactin stops the cell wall from performing processes that use the building blocks (lipids I, II, IIIWTA, or C55PP) as a reactant in a stepwise manner. This indicates that clovibactin does not stop enzyme function, but rather, it attaches itself to these reactants, indicating that these building blocks are the major target of the molecule [42].

Scientists use solid-state NMR to visualize how clovibactin engages with lipid II within the cell membranes [21], [30]. The antibiotic is radiolabelled to enhance its tracking. The interaction of clovibactin and llipid II results in clear ssNMR spectra of a stable clovibactin-lipid II complex. Clovibactin changes its conformation shape markedly when it bound to lipid II, suggesting it undergoes major structural alterations [21], [30], [42]. 

Coupled with ssNMR spectra, the confocal microscopic visualization of the clovibactin-lipid II complex elucidated large (supramolecular) structures [22]. The visualization showed that the anterior part of clovibactin became stiffer when it merged with lipid II, suggesting that it behaved like teixobactin, and further revealed giant unilamellar vesicles, or GUVs (balloon-like) structures, with large clusters of clovibactin-lipid II structures on their surface [22], [36]. 

## Analogues

The discovery of clovibactin is thrilling since it promises fresh ways to fight bacteria, but development and improvement may be sluggish, as it is difficult to produce [22]. Making clovibactin is very labour-intensive and yields only a minor quantity [22], [35]. Researchers are working on simpler versions of clovibactin and other depsipeptides to make production easier without losing its bactericidal action [40], [42]. Effective approaches to produce analogues may include methods that employ commercially available building blocks, require a mono-purification step, and produce a good yield. Clovibactin is one of the robust antibiotics that did not originate from conventional ribosomal protein-making processes and use D-amino acids. Other examples are teixobactin, vancomycin and polymyxin. Attention should be given to the high price of developing new antibiotics like clovibactin. Trial phases 1 and 3 may entail up to ten million dollars and a critical phase 3 trial can be five times as expensive [36], [46]. There are already several drugs commercially available to treat MRSA, e.g., linezolid, daptomycin, clindamycin, ceftaroline and doxycycline [47], [48]. A new drug, even one as auspicious as clovibactin, has to offer something unique to be worth its high price. In addition to inexpensive oral and intravenous options, there is also a growing market for injectable drugs (e.g., dalbavancin), which can be administered in a single dose and be effective for a lengthy period [49]. However, some new MRSA drugs cost thousands of dollars per dose, and hospitals are unwilling to add such expensive drugs to their already strained budgets [36].

When compared to MRSA, evidence suggests that there is more attention to developing new drugs to treat resistant gram-negative infections (e.g., *Klebsiella pneumoniae*). While there are many successful trials on Gram-negative infections, comparable studies for MRSA are wanting [50]. Clovibactin is a more recent discovery than malacidin and teixobactin, which are further along in development, but both face challenges as human trials are lacking. These new anti MRSA drugs are difficult to produce on a large scale and their development costs billions of dollars [46]. 

While these drugs are promising, incorporating these new products into an already full market is difficult. Weighing the costs and benefits of new antibiotics plays a significant role in aiding hospitals to choose the best antibiotics in terms of both patient care and funds. Finding less toxic and expensive derivatives of clovibactin effective against MRSA and even across the spectrum to Gram-negative bacteria would be ideal.

Needless to say, researchers are already trying to modify clovibactin to fashion an even more active compound. In the years ahead, these novel molecules will receive great attention. Healthcare providers often realise the limits of existing antibiotics, as many patients are not able to tolerate them.

**Figure 4:**
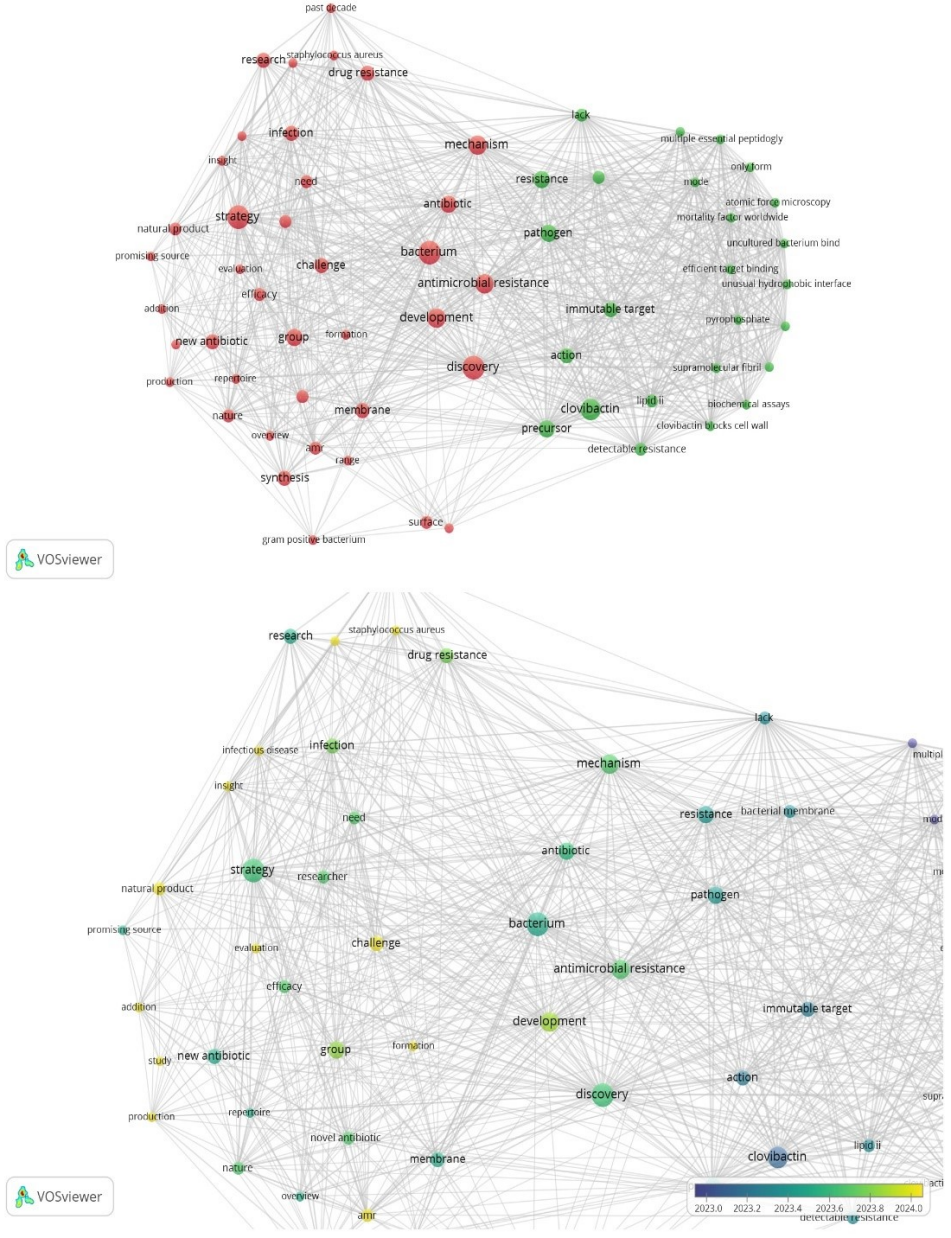
Current and future hot spot in clovibactin research

Future research (Figure 4 (Fig. 4)) on clovibactin will center on the use of innovative technology, such as fermentation processes and synthetic biology, to increase efforts and meet the necessities of hospitals and patients. It is of paramount importance to find new antibiotics, advance old ones, and produce them in new ways, mainly to target threatening bacteria such as *S. aureus* and its resistant strains. 

The scientific community strives to combat diverse diseases by determining how antibiotics function and producing better treatments. A good illustration of this is the increasing understanding of the new paths by which bacteria obtain resistance to drugs and discovering new means to stop or slow these phenomena. Work is being done on designing new drugs to develop safer, non-toxic antibiotics that are effective against both metabolically active and dormant bacteria using technologies, such as deep learning, generative AI, metagenomics, iChip technology, synthetic biology, CRISPR-Cas9 screening, high-throughput screening, exploring extremophiles, and phage therapy and lysins. Critically assessing past achievements and disappointments in antibiotic discovery will help advance future research. Another thematic focus is biofilms and searching for ways to destroy them for better therapeutic outcome. 

Overcoming hurdles such as stringent regulations, costs, and the labour-intensive development of novel drugs requires interdisciplinary collaboration and new methodologies.

## Conclusion and future directions

The antibiotic clovibactin is a new depsipeptide which is highly efficacious against *S. aureus* and its drug-resistant strains. It represents a ground-breaking advancement in antibiotic discovery. Its molecular structure, unique mechanism of action and bactericidal ability (targeting the lipid II biosynthesis pathway) make it a good choice as a promising candidate for developing new antibiotics and treating unresponsive infections. Future research work should focus on design and synthesis of highly potent clovibactin analogues against *S. aureus*, methicillin-resistant (MRSA) and vancomycin-resistant (VRSA) *S. aureus*; total synthesis of clovibactin; the use of clovibactin as a scaffold for unlimited new antimicrobial peptides; in-vitro antibacterial activity of clovibactin and possible analogues against MRSA and other clinical bacterial Isolates; further investigations into its killing mechanism against MRSA; and exploring the likelihood of de novo resistance to clovibactin. Continued research and innovative approaches are essential to safeguard these vital medicines and address the growing threat of antibiotic resistance.

## Notes

### Author’s ORCID

- Adeiza Shuaibu Suleiman: 0000-0002-9293-2600

### Funding 

None.

### Competing interests

The author affirms that he has no competing interests.
